# Sex differences on laser-induced choroidal neovascularization and short-chain fatty acid treatment in a mouse model

**DOI:** 10.1186/s12974-025-03508-1

**Published:** 2025-07-19

**Authors:** Chufan Yan, Caio Andreeta Figueiredo, Inga-Marie Pompös, Bilge Ugursu, Paula Arribas-Lange, Sergej Skosyrski, Seulkee Yang, Petra Althoff, Norbert Kociok, Antonia M. Joussen, Susanne A. Wolf

**Affiliations:** 1 Department of Ophthalmology, Charité – Universitätsmedizin, Berlin, Germany; 2 Psychoneuroimmunology, Max-Delbrück-Center for Molecular Medicine in the Helmholtz Association, Berlin, Germany

**Keywords:** Age-related macular degeneration, Choroidal neovascularization, Retinal microglia, Short-chain fatty acids, Sex differences

## Abstract

Age-related macular degeneration (AMD) is a leading cause of blindness worldwide, with a clinical presentation that varies between sexes. In late-stage AMD, choroidal neovascularization (CNV) triggers retinal inflammation and degeneration, processes that are exacerbated by an overactive response of retinal microglial cells. Short-chain fatty acids (SCFAs) have emerged as potential treatments for AMD due to their anti-inflammatory properties. In this study, we investigate the effects of SCFA treatment in a laser-induced CNV mouse model, focusing on sex-dependent differences in disease progression and microglial response. Our findings demonstrate distinct sex-specific patterns in the development of CNV and associated pathological hallmarks. SCFA treatment resulted in a slight increase in density of Iba1^+^ microglial cells in females at 3 days post-laser (3dpl), while it prevented an increase in males at 7 dpl, with both sexes showing enhanced microglial ramification. The dynamics of microglial density were likely linked to protective effects on CNV lesion, leakage size, and inflammation, which occurred earlier in females and later in males. At transcriptional level, SCFA showed mixed effects, mainly targeting inflammation resolution, mitochondrial support, and neuronal repair in a sex-dependent manner. In vitro, SCFAs reduced microglial phagocytosis of retinal debris, suggesting a potential anti-inflammatory action. This study underscores the importance of considering sex-specific responses in the development of AMD treatments, such as SCFAs, and highlights the need for personalized therapeutic strategies.

**Supplementary Information:**

The online version contains supplementary material available at 10.1186/s12974-025-03508-1.

## Introduction

Age-related macular degeneration (AMD) stands as a prevalent and debilitating eye disease, and one of the leading causes of permanent visual impairment worldwide [1]. AMD’s prevalence and clinical manifestations may vary between male and female individuals [2, 3], and epidemiological studies reported a higher risk of AMD in females compared to males [4, 5]. Still, only a few studies have considered sex differences in ocular diseases, leaving AMD’s sex-specific disparities poorly defined [6] AMD’s pathology is characterized by the accumulation of subretinal deposits of lipids, proteins, and cellular debris, termed drusen bodies, that induce inflammation in the retina [7, 8]. Retinal microglia, the local resident immune cells, respond to this accumulation and trigger phagocytosis of retinal components in close contact with drusen bodies, such as photoreceptors and supportive glial cells, thus potentially jeopardizing vision [7, 8]. Moreover, continuous reactive state of retinal microglia induces recruitment of other inflammatory immune cells to the subretinal space, further intensifying neuroinflammation in the eyes [9, 10].

A significant late complication of AMD is the choroidal neovascularization (CNV), a hallmark lesion of advanced neovascular AMD (nAMD, or also called wet-AMD), characterized by aberrant blood vessel formation, which causes exudation, hemorrhage, and consequently retinal fibrosis and degeneration [11–15]. Anti-vascular endothelial growth factor (anti-VEGF) therapy is a standard treatment for nAMD, but its efficacy varies among patients, and a need for alternative or supplementary treatments targeting retinal inflammation has become imperative [16, 17]. Recent research has shown the involvement of gut microbiota in ocular health and disease, and the concept of a gut-retina axis has gained more attention in the field [18]. For example, patients diagnosed with AMD and/or diabetic retinopathy have shown altered intestinal microbiota (i.e. gut dysbiosis) [19–21]. Short-chain fatty acids (SCFA), bacterial metabolites derived from dietary fiber fermentation, have promised benefits in mitigating retinal inflammation [20, 22]. Thus, here we investigate the effects of SCFA supplement treatment in a laser-induced CNV mouse model of nAMD. Moreover, we explored baseline sex-specific differences in this model, and how SCFA treatment affects males and females differently.

## Methods

### Animal husbandry

C57BL/6J mice were purchased from Janvier (Cedex, France) and housed in Charité animal houses. 6 to 9-week-old mice were used in this study. Mice were provided with unrestricted access to food pellets and water *ad libitum*, and were group-housed in ventilated cages under standard laboratory conditions (12:12 light/dark cycle with light on at 6:00 h, 22 ± 2 °C, 45–60% humidity). All animal experiments complied with the guidelines of the *ARVO Statement for the Use of Animals in Ophthalmic and Vision Research* and were approved by the local governmental authorities (*Landesamt für Gesundheit und Soziales*, LaGeSo, Berlin, Germany: G0056/20).

### Mouse model and group design

Nine-week-old mice were anesthetized with subcutaneously injections of ketamine (100–120 mg/kg) (100–120 mg/kg; CP-Pharma, Germany) and xylazine (10–15 mg/kg; CP-Pharma), and had their pupils dilated with 5% phenylephrine-tropicamide eye drops (Charité Apotheke, Berlin, Germany). Optimal pupil dilation (2.5–3 mm) was confirmed using an ophthalmological slit lamp. To flatten the cornea, a round glass coverslip (15 mm diameter) was used as a contact lens, and it was placed on the mouse cornea with lubricating eye drops, Methocel^®^ (OmniVision, Germany) in a perpendicular position to the laser beam. Using the image-guided laser system VISULAS^®^ 532s (Carl Zeiss, Germany), the focus laser beam was positioned on the choroidal retinal pigmented epithelial (RPE) cell layer on the eye fundus, and four laser burn-spots around the optic nerve were induced one by one in each eye. The system was equipped with an argon laser with a wavelength of 532 nm, pulsed with a fixed diameter of 50 μm, duration of 100 ms, and 120 mW of intensity. After that, the eyes were gently rinsed with Corneregel^®^ (Bausch + Lomb, Germany) to protect them from corneal opacification owing to dehydration [23]. The mice were then placed in their cages with hot water bottles or a red lamp to keep them warm until they awoke. To allow accurate evaluation of the laser-induced CNV lesions, some animals were excluded from the study based on (1) presence of hemorrhages, and (2) bleeding area diameter. Severe hemorrhages lead to larger CNV lesions, with outcomes that cannot be compared to non-hemorrhagic eyes. Moreover, lesions with a bleeding area diameter less than that of the lesion were considered Grade 0 for analysis. Lesions with a bleeding area diameter larger than the lesion but less than two times the lesion diameter were excluded from quantification (Grade 1). All lesions in the same eye were excluded from analysis if the bleeding area diameter was more than two times the lesion diameter (Grade 2). Lesion areas were defined based on IR images from the first analysis day (3dpl), and closely located lesions were considered in the analysis. What appears to be lesion fusion on FA or flat-mount images often reflects leakage or proximity but not actual anatomical fusion of laser sites. IR images—used to define ROIs—were carefully evaluated to exclude fused lesions. Fused lesions (i.e., lesions with adjacent lesion cores) were excluded from the analysis.

SCFA was administered to mice *ad libitum* via drinking water, and the SCFA concentrations of choice were similar to the ones previously described [24, 25]. 67.5mM sodium acetate (5.54 g/L, Cat#S2889-250 g, Sigma-Aldrich, USA), 25mM sodium butyrate (4.4 g/L, Cat#303410–100 g, Sigma-Aldrich, USA), and 40mM sodium propionate (2.48 g/L, Cat#P1880-500 g, Sigma-Aldrich, USA) were prepared and diluted with tap water under sterile conditions, followed by sterile filtration with Steritop^®^ filters (Cat#S2GPT02RE, Merk Millipore, USA). All the materials used in this process were autoclaved to avoid contamination and ensure maximum SCFA stability. The drinking SCFA solution was stored at 4ºC for no more than 7 days, and was changed every 48–72 h [25, 26].

C57BL/6J mice were randomly divided into two groups: the control group received only the laser lesion without SCFA supplementation, and the SCFA-treated group received SCFA in drinking water 3 weeks before the laser lesion until the mice were euthanized and samples were collected. All the animals included in this study underwent the same standardized experimental procedures. The retina and choroid were collected at 3-, 7-, and 14-days post-laser induction (dpl) for both sex groups and prepared for subsequent experiments.

### Estradiol serum analysis

Blood samples were collected from mice at the time of sacrifice via cardiac puncture. After clotting at room temperature, samples were centrifuged at 2,000 × g for 10 min to obtain serum. Estradiol concentrations were determined in 50 µl serum using a Mouse Estradiol Rapid ELISA Kit (Catalog #EELR013, Invitrogen), according to the manufacturer’s instructions. All samples were analyzed in duplicate.

### Ocular imaging analysis (IR, BAF, FA)

Mice were anesthetized and their pupils dilated with 5% phenylephrine-tropicamide eye drops (Charité Apotheke, Berlin, Germany) before being positioned horizontally on a polystyrene box attached to a head holder. Infrared reflectance (IR) and BluePeak blue laser autofluorescence (BAF) imaging were acquired using the 55° Wide Field Lens. The Automatic Real Time (ART) Mean function was active during the acquisition, and the “compute mean” function was applied after acquisition. For Fluorescein Angiography (FA), mice received an intraperitoneal injection of 5 µg/g body weight of fluorescein Alcon 10% dye (Alcon, Germany). Images were acquired 6 min post-injection using Spectralis HRA + optical coherence tomography (OCT) (Heidelberg Engineering, Heidelberg, Germany) and managed with Heidelberg Eye Explorer software (version 1.7.0.0). IR, BAF, and FA data were exported in “.tif” format and further analyzed using ImageJ software (v2.14.0/1.54 h, National Institutes of Health, USA). Circular region-of-interest (ROIs) were overlapped with lesions and set according to IR images from the first analysis day (3dpl). Recordings from different days were aligned using the optic nerve head (ONH) position and retinal vessels. Then the lesion ROIs were overlapped, keeping the same relative position to the ONH. Alignments were performed in ImageJ using the “transform & translate” function. Mean gray values intensity (MGVI) from each ROI, in each parameter (IR, BAF, FA) were obtained.

### RNA sequencing sample preparation and cDNA library construction

Each RNA sample was a pool of two laser induced retinas extracted from mice on the same group, using the RNeasy Mini kit (Cat#74104, Qiagen, Germany) and sent to Novogene Co, Ltd. (Munich, Germany) for RNA sequencing. Briefly, the integrity, purity and initial quantitation of RNA samples were measured using the Bioanalyzer 2100 (Agilent Technologies, USA). The Novogene NGS RNA Library Prep Set (PT042) was utilized for library construction. Quality assessment of the constructed library was performed with Qubit 2.0 (Thermo Fisher Scientific, Germany) and RT-PCR. We started out with 6 samples per group. We had to pool 2 retinae from two different animals per n – information of 6 independent samples went into the group analysis in total. Due to quality assessment, the number of n in some of the female groups was reduced from 3 to 2 (for 7- and 14 dpl, male-control *n* = 3, male-SCFA *n* = 3; for 7 dpl, female-control *n* = 2; female-SCFA *n* = 2; for 14 dpl, female-control *n* = 2; female-SCFA *n* = 3 (see the PCAs in supplementary material 5 A-D). Quantified libraries were then pooled and sequenced on the Illumina Novaseq 6000 platform using a paired-end 150 strategy. The data was reported as raw read counts and fragments per kilobase of transcripts per million. DEseq2 was used to calculate the normalized read counts. Differentially expressed genes (DEGs) were identified with a fold-change > |1| and adjusted *p* value (padj) < 0.1. We choose this p value aiming to include low abundance microglia-related genes. Principal Component Analysis (PCA) plots were done using DESeq2 (R/Bioconductor); Heatmaps and volcano plots were generated using the PTMCloud tools (https://www.ptm-biolab), and Gene Ontology (GO) Analysis was done using David (https://david.ncifcrf.gov) (Supplementary material 5E-H, full list Supplementary material 6). Due to ethical constraints and the lack of additional biological material, qPCR-based validation of the RNA-seq results was not feasible; we therefore interpret the transcriptomic findings with appropriate caution and consider them exploratory in nature. The raw and processed RNA-seq data generated in this study have been deposited in the NCBI Gene Expression Omnibus (GEO) under accession number GSE299691.

### Flat mount preparation

Mice were sacrificed by cervical dislocation, followed by enucleation of the eyes. The eyes were initially immersed in a 4% paraformaldehyde (PFA) solution in Dulbecco’s phosphate-buffered saline (DPBS, Cat#14190169, Thermo Fisher Scientific, Germany) for 15 min at room temperature (RT), and then transferred to DPBS for further processing. The retinas were isolated and cut into four-leaf shapes under a stereomicroscope. Samples were stored in methanol at -20 °C until further use.

### Immunofluorescence of retinal and choroidal flat mounts

The samples were permeabilized with tris-buffered saline (TBS, Cat#498276376, Carl ROTH, Germany) 5% Triton X-100 at RT for 15 min, followed by 1h blocking with 1% Triton X-100 in blocking buffer (10% donkey serum in TBS). Next, samples were incubated with anti-Iba1 antibody solution (dilution 1:250, Cat#ab5076, Abcam, UK) and Isolectin GS-IB_4_ conjugate with Alexa Fluor™ 647 (dilution 1:250, Cat#I32450, Invitrogen, USA) for 2 days at 4 °C. The samples were rinsed three times at RT for 10 min in TBS, and then incubated with Alexa Fluor 488 AffiniPure™ Donkey Anti-Goat IgG (1:200 dilution, Cat# 705-545-147, Jackson ImmunoResearch, USA) in blocking buffer (2 h, 4 °C) followed by DAPI nuclear staining for 10 min at 4 °C (10 µg/ml in TBS, Cat# D9542, Sigma-Aldrich, Germany). After washing (3x TBS, 10 min each) samples were mounted with Aqua-Poly/Mount (Cat#18606, Polysciences, USA) on glass slides, and stored at 4 °C until dry-out. Images were acquired at the middle point between the optic nerve head (ONH) and the end of the flat mount leaf, neighboring the laser-lesion area but not including it. Unfortunately, strong autofluorescence, signal saturation, and frequent tissue disruption at the lesion core made consistent quantification unreliable. To avoid bias from selectively preserved lesions, we chose peri-lesion regions for reproducibility. Z-stacks from the full depth of each imaged area were acquired with a confocal microscope, and images were divided into ILP and OLP series according to the vessel size (IPL, large vessels; OPL, small vessels). Images were further analyzed using ImageJ software (version 2.14.0/1.54 h), and the microglial morphology analysis was performed following the protocol provided by Young et al. 2018 [27]. 1–2 maximum intensity projections from each retinal layer were analyzed, per retina, with 3–5 animals per group and per time-point. Cell density values were further divided by the image area, and presented as cells/mm². For choroid flat mount staining, only Isolectin GS-IB_4_ Alexa Fluor™ 488 Conjugate (dilution 1:250, I21411, Invitrogen™, USA) was used, and images were acquired with a confocal microscope at laser spots, with Z-stacks from the full depth of the choroid.

### SCFA in vitro treatment

To perform SCFA treatment in vitro, a 100x SCFA stock solution was prepared in MilliQ-grade water consisting of 30mM acetate (Cat# S2889), 2mM of propionate (Cat# P5436) and 1mM butyrate (Cat# 303410), all purchased from Sigma-Aldrich^®^ (Merck, Germany). The final solution was sterile filtered at 0.22 μm (Cat# FCA206030, JetBiofil Europe, Spain), aliquoted and stored at -20 °C until further use. For the experiments, the stock solution was diluted to 1x to obtain final concentrations of 300µM, 20µM, and 10µM, (acetate, propionate, butyrate) respectively.

### Human induced pluripotent stem cells (hiPSC)

Human induced pluripotent stem cells (hiPSC) from a specific cell line (BIHi043-A, subclone HMGUi001-A-10 that express GFP (XM001-AAVS1-GFP-Cl10)) were obtained from the Max Delbrück Center (MDC) Technology Platform Pluripotent Cells facility at the MDC Berlin-Buch Campus. All experiments were conducted on this facility, following their standardized protocols described elsewhere [28].

Briefly, hiPSC clusters were cultivated at 37 °C, 5% CO_2_ on Geltrex-coated plates (0.12–0.18 mg/ml, Gibco™, ThermoFisher Scientific), with StemMACS iPS-Brew XF medium (Miltenyi Biotec, Germany), split weekly using StemPro Accutase^®^ (Gibco™, ThermoFisher Scientific), and supplemented with 0.5 µM thiazovivin (STEMCELL Technologies, Canada) for the first 24 h after split. hiPSC clusters were further used to obtain human microglia-like (hiMGL) cells.

### Generation of human microglia-like (hiMGL) cells from hiPSCs

The hiMGL cells were generated from differentiated hiPSC, as previously described [28–30]. Firstly, hiPSC clusters were differentiated into hematopoietic progenitor cells (HPCs) in Geltrex-coated plates using the STEMdiff™ hematopoietic kit (STEMCELL Technologies). After 12-days of differentiation, HPCs were split and seed at 1.2 × 10^5^ cells/well of a six-well plate) into DMEM/F12 (w/o Phenol Red)-based serum-free microglia differentiation medium with B27 (2×), insulin-transferrin-selenite (ITS, 2×), N-2 (0.5×), GlutaMAX (1×), MEM non-essential amino acids solution (1×) (all purchased from Gibco™, ThermoFisher Scientific), insulin (5 µg/ml; PromoCell, Germany) and α-thioglycerol (400 µM; Merck, Germany). Additionally, IL-34 (100 ng/ml; Peprotech, USA), TGF-β1 (50 ng/ml; Peprotech) and M-CSF (25 ng/ml; Peprotech) were supplemented in the medium every other day. On day 25 and day 27 post HPC split, CD200 (100 ng/ml; Novoprotein America, USA) and fractalkine (CX3CL1, 100 ng/ml; Peprotech) were added to the medium (i.e., maturation medium) to support microglial maturation. Finally, hiMGL cells were harvested between days 28 and 31 post HPC split, and seeded to the phagocytosis assay.

### Müller cell debris solution

The human Müller cell line Moorfields/Institute of Ophthalmology- Müller 1 (MIO-M1) was obtained from the UCL Institute of Ophthalmology (London, UK) by the Department of Ophthalmology, Charité – Universitätsmedizin (Berlin, Germany). MIO-M1 cells were grown in high glucose DMEM containing 10% FBS and 1% Pen/Strep until confluence, and incubated at 37 °C, 5% CO_2_. Cells were detached from culture vessels with StemPro Accutase^®^ (Gibco™, ThermoFisher Scientific), rinsed (400× g, 10 min, 4 °C) and re-suspended in DPBS. Cell suspension was dounce homogenized 20–30 times with a tight pestle, and three frozen/thaw cycles were performed to rupture cellular membranes. To label Müller cells debris with pHrodo™Red, the solution was spun down (20,000× g, 5 min, 4 °C), and the pellet re-suspended in 0.1 M sodium carbonate (Na_2_CO_3_, Cat#A135.1, Carl ROTH, Germany) containing pHrodo™Red succinimidyl ester (1:140; Cat# P36600, ThermoFisher Scientific), and incubated (1.5 h, 37 °C, 1,000 rpm) protected from light. Then, the solution was spun down (20,000× g, 5 min, 4 °C), and the pellet was washed three times by adding 1.5 ml DPBS and spinning down (20,000× g, 5 min, 4 °C). Final Müller cell debris solution obtained was 200 µl, aliquoted and stored at -70 °C until further use. All procedures were performed under sterile conditions.

### Retinal debris solution

Retinas from C57BL/6J female mice were isolated, dissected under a stereo microscope, and placed in ice-cold DMEM medium. Eight retinas were pooled in MACS buffer (PBS, 0.5% BSA, 2mM EDTA, at pH 7.3), and dounce homogenized 15–20 times with a tight pestle. The cell suspension was passed through a 100 μm filter (Cat#130-098-463, Miltenyi Biotech, Germany). To discard microglia and other myeloid cells from retinal debris, the cell suspension was incubated (15 min, 4 °C) with anti-mouse CD11b^+^ magnetic beads (1:40; Cat#130-049-601, Miltenyi Biotech), and then passed through pre-wet LS MACS columns (Cat#130-042-401, Miltenyi Biotech) attached to a QuadroMACS™ Separator (Cat#130-091-051, Miltenyi Biotech). The flow through with additional 0.5 ml of MACS buffer was collected, and three frozen/thaw cycles were performed to rupture cellular membranes. To label retinal debris with pHrodo™Red, the solution was spun down (20,000× g, 5 min, 4 °C), and the pellet re-suspended in 0.1 M sodium carbonate (Na_2_CO_3_) containing pHrodo™Red succinimidyl ester (ThermoFisher Scientific), and incubated (1.5 h, 37 °C, 1,000 rpm) protected from light. Finally, the solution was spun down (20,000× g, 5 min, 4 °C), and the pellet was washed three times by adding 1.5 ml DPBS and spinning down (20,000× g, 5 min, 4 °C). Final retinal debris solution obtained was 400 µl, aliquoted and stored at -70 °C until further use. All procedures were performed under sterile conditions.

### BV-2 cell culture

Murine BV-2 cells were grown at 37 °C, 5% CO_2_ until 80% of confluence in complete culture medium, i.e., high glucose Dulbecco’s Modified Eagle Medium (DMEM), containing 10% fetal bovine serum (FBS) and 1% Penicillin/Streptomycin (Pen/Strep). Cells detached from culture vessels with StemPro Accutase^®^, rinsed (400× g, 10 min, 4 °C) and re-suspended in cultivation medium. Cells were seeded according to the assay, or cultivated for further use. All products were purchased from Gibco™, ThermoFisher Scientific.

### Primary neonatal microglia

Primary microglia culture was obtained from 7 to 10 C57BL/6J pups (P1 to P3) males and females brain tissue per preparation. Briefly, cerebellum and olfactory bulb were discarded, and tissue from each sex group pooled, rinsed abundantly with HBSS (Cat#14175-053, Gibco™, ThermoFisher Scientific), digested for 2 min at RT with 6.67 mg/ml trypsin (Cat# L2103-20, Biochrom AG, Germany) and 0.34 mg/ml DNase I in HBSS (Cat# #LS002139, Worthington Biochemical, USA). The digestion stopped by addition of 5 ml complete medium (DMEM, 10% FBS, 1% Pen/Strep), and the tissue was mechanically disrupted using a glass Pasteur pipette in the presence of 2.5 mg/ml DNase I in HBSS. Next, 10 ml of complete medium were added, samples spun down (128× g, 10 min, 4 °C), the pellet re-suspended in complete medium, and cells spread in 4–5 pre-coated T75 culture flasks. Flasks were previously pre-coated for 30 min using 0.1 mg/ml poly-l-lysin hydrobromide (PLL, Cat#P1274, Sigma-Aldrich, Germany) solution in distilled water, and then abundantly rinsed with DPBS before receiving the cell suspension. The suspension was cultivated initially for 2 days in complete medium, had the medium exchanged, and was grown for a further 7 days. To induce microglia proliferation and differentiation, the medium was exchanged with a complete medium supplemented with L929 conditioned-medium (1:3 in volume) on day 9. On day 11, microglial cells were enriched on the surface of the culture, and were shaken off using an orbital shaker (150 rpm, 30 min, 37 °C). Cells were pooled from all flasks, spun down (129× g, 10 min, 4 °C), and cell number determined using Trypan blue.

### Phagocytosis assay – Incucyte^®^ Live-Cell analysis system

For hiMGLs, cells were seeded at 5 × 10^4^ cells/well (96 well/plate) in the previously referred maturation medium. After a resting period of 8 h at 37 °C, 5% CO_2_, cells were treated with SCFA solution, and after 4 h the pHrodo-labeled Müller cells debris were added at 1:80 dilution per experimental condition. The assay was further incubated and monitored for 12 h inside an incubator (37 °C, 5% CO_2_) equipped with the Incucyte^®^ SX5 Live-Cell Analysis System (Sartorius BioAnalytical Instruments Inc., Germany).

For BV-2 cells and primary microglia, cells were seeded in DMEM 1% Pen/Strep medium at 5 × 10^4^ and 2 × 10^4^ cells/well (96 well/plate), respectively, in quintuplicates per group (control or SCFA), per condition (pHrodo™ Red E. coli BioParticles™ or retinal debris), and per sex group (primary microglia). After 1 h resting period, cells were treated with SCFA solution, and control groups received only medium. After 4 h of initial incubation (at 37 °C, 5% CO_2_), pHrodo™-labelled retinal debris solution (at 1:80 dilution per experimental condition) or pHrodo™ Red E. coli BioParticles™ (1:500 from 1 mg/ml stock solution, Cat# P35361, ThermoFisher Scientific) were added. The assay was further incubated, and pHrodo™ fluorescence was monitored using Incucyte^®^ every 1 h or 30 min for 16 h. Independent of cell type, four images/well were taken using 20x objective (phase contrast and orange channel, Ex/em = 556/609nm; acquisition time = 400ms). The analysis definition was determined for each channel: for phase contrast channel, Segmentation = AI Confluence; Cleanup adjust size = -3; Filter area min = 100.00 µm^2^. For orange channel, Segmentation = fixed threshold (threshold OCU = 1.3), Edge Split Off; Filter area min = 20.00 µm^2^. The parameters “focus” and “confluence” were used to identify image artefacts and exclude them from the analysis. The readouts were extracted as total pHrodo integrated intensity (OCU x µm^2^/image), and plotted over time (h).

### Phagocytosis assay – Flow cytometry

hiMGLs phagocytic capacity was further analyzed via flow cytometry. Cells were preincubated with SCFA solution for 4 h, then the pHrodo-labeled Müller cells debris was added (1:80 dilution) per experimental condition, and the assay further incubated for 12 h inside an incubator (37 °C, 5% CO_2_). After that, supernatants were discarded, and hiMGLs detached from wells using StemPro Accutase^®^ (10 min, 37 °C), rinsed (400× g, 10 min, 4 °C) and re-suspended in FACS buffer (DPBS, 2mM EDTA,10mM HEPES, 2% FBS). For staining, cells were incubated with Human BD Fc Block™ (clone Fc1, RUO, BD Biosciences, Germany) for 20 min at 4 °C, and further stained (30 min, 4 °C) with fluorochrome-conjugated antibodies: CD45 (eFluor450; clone 2D1; 1:50; Cat#48-9459-41, eBioscience™) and CD11b (PE-Cy7; clone ICRF44; 1:100; Cat#301321, BioLegend). DAPI (1:1000, Cat#32670; Merck) was added as viability dye. Finally, cells were washed (400× g, 5 min, 4 °C) and re-suspended in FACS buffer. Samples were immediately acquired using LSRFortessa (BD Life Sciences, USA), and further analyzed with FlowJo™ (v10, LLC, BD Life Sciences, USA).

### Statistical analysis

Results were statistically analyzed using GraphPad Prism 7 (GraphPad Software Inc., USA), post-test corrections were applied according to software recommendations. All results were given as mean ± SEM, or as mean ± SD, and are representative of at least two independent experiments. For ocular imaging analysis, each lesion was taken as one data point, and treated as repeated measurements (same lesions over different time points). One-way and two-way ANOVA were used in the data, with assumed sphericity and alpha = 0.05 (*p* ≤ 0.05.), and are indicated in the figure legends. For phagocytosis analysis using flow cytometry (FACS), data were analyzed by unpaired *t*-test (two-tailed). Statistically significant values are indicated by asterisks or # in each figure. In addition, we performed a mixed model analysis, mainly to evaluate whether we can treat eyes from the same animal as independent samples.

### Statistical analysis for ocular imaging parameters using a mixed model

We applied mixed model statistics to evaluate:

- Whether both eyes of one mouse can be used as independent samples.
- The impact of sex on the parameters.
- The impact of treatment on the parameters.

Additionally, we adjusted the analysis strategy for FA due to convergence issues.

We implemented a nested random effects model, where “Eye” was nested within “Animal ID,” to assess the independence of measurements from both eyes of the same mouse. The variance component for “Eye” was substantial, indicating that measurements (IR and BAF) from different eyes of the same mouse varied significantly. Since each eye contributed distinct variability, we treated them as independent samples without violating statistical assumptions or inflating type I error rates. For the FA, the initial model failed to converge, but after transformation and scaling, the model did converge, again showing substantial variance at the eye level.

While FA required a different statistical approach, the results still support the conclusion that each eye can be treated as an independent measurement. Thus, the mixed model confirms that both eyes can be considered independent samples rather than requiring a strict within-animal pairing. Sex was included as a fixed effect in the models, and treatment (SCFA) was additionally included as a fixed effect.

The mixed model supports treating both eyes as independent samples, as substantial between-eye variability was detected. Sex significantly influenced IR and FA but not BAF. SCFA treatment significantly affected IR but had no effect on BAF or FA. FA required a different model due to convergence issues, highlighting the need for data transformations and alternative statistical approaches.

## Results

### SCFA modified CNV injury progression in a sex-specific manner

**Fig. 1:**
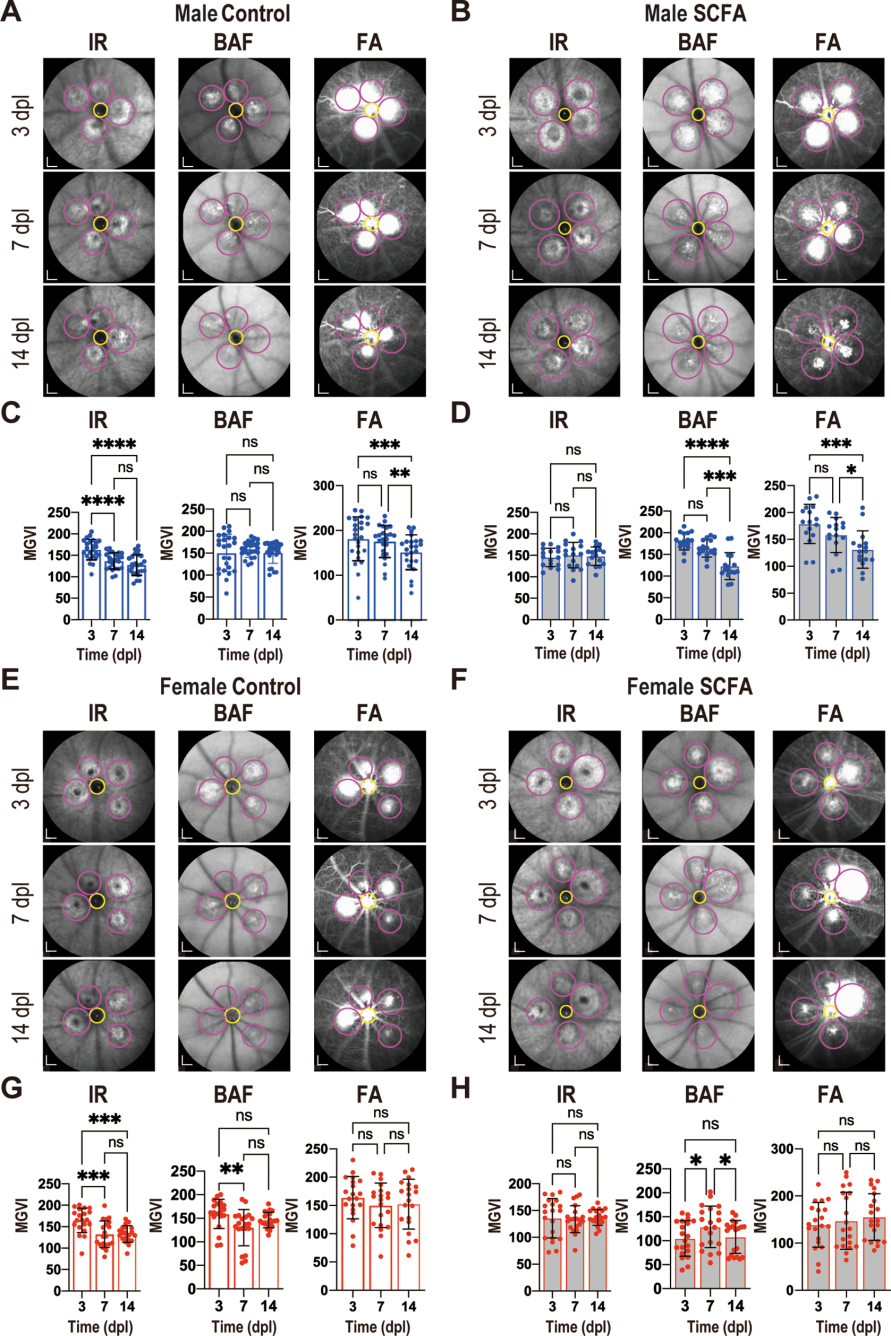
Ocular imaging and lesion-oriented analysis *in vivo.* After CNV laser-induced injuries, anesthetized animals were examined over time by ocular analysis of near-infrared imaging (IR), blue-laser fundus autofluorescence (BAF), and fundus angiography (FA). (**A**,** B**,** E**,** F**) Ocular images were recorded at 3-, 7- and 14-days post-laser (dpl) across groups. Laser lesions were identified at 3dpl images (magenta circles), and used as regions of interest (ROIs) monitored over time. The optic nerve head (ONH) was identified (yellow circle) and used to align images from different days. (**C**,** D**,** G**,** H**) Mean gray values intensity (MGVI) was quantified – from the ROIs using ImageJ. Bar charts show individual lesion-oriented values and mean ± SD, *n* = 16–24 lesions, *p < 0.05, **p < 0.01, ***p < 0.001. ****p < 0.0001 (one-way ANOVA, Tukey’s multiple comparisons test); scale bar = 1 mm

To evaluate the therapeutic effects of SCFAs in nAMD in vivo, a widely accepted laser-induced CNV mouse model was utilized, as its relevance for nAMD studies has been previously shown [23]. Animals were randomly assigned to either the control or treatment group, with SCFA solution continuously administered for three weeks before injury induction (Supplementary material 1 A). This preventive SCFA treatment aimed to mimic SCFA derived from intestinal microbiota upon dietary fiber intake, which has been strongly linked to reduced inflammation and is widely recommended in clinical practice [31]. In preliminary experiments, SCFA was administered under different conditions: one week before laser lesion (continued for two weeks), on the day of the lesion, and one week after it. The preventive setting—starting SCFA administration one week prior—demonstrated the most potent effects in in vivo assays (Supplementary material 1B-C). Based on these findings, we selected the pre-treatment regimen for further investigation, and continued it for 3-, 7-, or 14-days post-laser lesion (dpl). To monitor injury progression, ocular images were collected, followed by lesion-oriented analyses using near-infrared imaging (IR), blue-laser fundus autofluorescence (BAF), and fundus angiography (FA) (Fig. 1).

IR imaging revealed characteristic hyper-reflective CNV lesions in the retinal pigmented epithelial (RPE) cells of the choroid, serving as a readout for lesion development. When using the mixed model statistical analysis on all data converged, a significant effect of sex was found (*p* = 0.027), with male mice exhibiting higher IR values, and SCFA treatment had a significant effect (*p* = 0.038), suggesting a treatment-related change in IR. Over time, hyper-reflectiveness decreased in untreated groups (Fig. 1A, C, E, G), whereas CNV lesions remained stable in SCFA-treated males and females over time (Fig. 1B, D, F, H). Notably, SCFA reduced lesion hyper-reflectiveness at 3 dpl in females only, suggesting a smaller CNV (Supplementary material 2 A).

BAF analysis was used to assess inflammatory infiltration in lesion areas. When taking all data together and applying a mixed model analysis, no significant effect of sex and treatment was observed. However, when analyzing males and females separately, in untreated animals, no difference was detected over time in males, whereas in females, infiltration decreased from 3 to 7 dpl (Fig. 1A, C, E, G). Upon SCFA treatment, BAF decreased over time in males, whereas in females BAF slightly increased from 3 to 7dpl and decreased again from 7 to 14dpl (Fig. 1B, D, F, H). Comparing treated and untreated animals for each time point, SCFA decreased BAF at 14dpl in males. In females, the decrease in BAF was evident at 3dpl and 14dpl and was significantly more pronounced than in males (Supplementary material 2B). Overall, SCFA-treated females displayed significantly lower inflammatory infiltration over the whole period than untreated females and untreated and treated males.

Due to model convergence issues, the mixed model applied for IR and BAF failed to converge for FA (assessing retinal vascular leakage), likely due to high variability and non-normality in the FA data distribution. To address this, we log-transformed the FA data and applied a Box-Cox transformation to stabilize variance. A scaled version of the transformed data was used, which improved model convergence. With this modified approach, the model for FA successfully converged, revealing a significant effect of sex (*p* = 0.009) but no significant effect of treatment or time when all data were collectively analyzed. When separating males and females, the FA analysis showed males with reduced vascular leakage up to 14 dpl, regardless of SCFA treatment (Fig. 1). At 3dpl, treated females displayed a lower FA compared to treated and untreated males at the same time point (Supplementary material 2 C).

Additional information on CNV size was depicted with choroidal flat mounts stained with Isolectin-B4, although quantification of CNV volume was not possible due to inconsistencies in the correct CNV area definition (Supplementary material 3).

### Sex-specific effects of SCFA treatment on retinal inflammation in laser-induced CNV mouse model

**Fig. 2:**
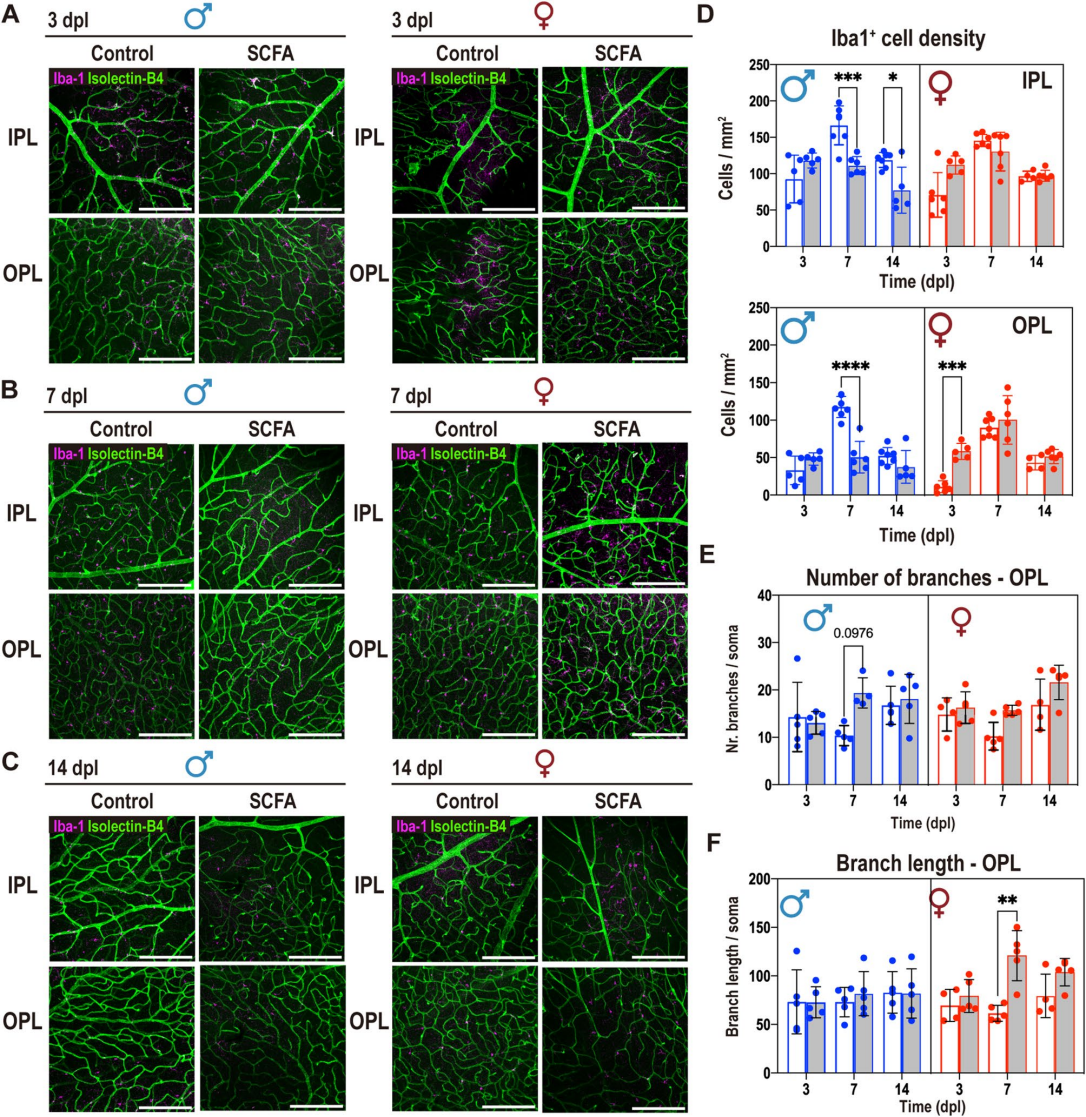
Effects of SCFA treatment on Iba1^+^ cell dynamics. Images were acquired at the middle point between the optic nerve head (ONH) and the end of the flat mount leaf, neighboring but not including the laser-lesion area. Technical limitations—such as tissue overlap and strong signal saturation—prevented reliable lesion-core quantification. Immunofluorescence of retinal flat mount identifying Iba1^+^ cells (purple) in two distinct retinal layers, IPL (upper row) and OPL (lower row), identified by retinal blood vessels (Isolectin B4^+^, green). (**A**) Representative images from 3dpl male and female retina. (**B**) Representative images from 7dpl male and female retina. (**C**) Representative images from 14dpl male and female retina. (**D**) Iba1^+^ cell density in IPL, and in OPL over time 3-14dpl; data show individual values and mean ± SD, *n* = 4–7, *p < 0.05, *** *p* < 0.001, **** *p* < 0.0001 (2-way ANOVA, Tukey’s multiple comparisons test). (**E**) Quantification of number of branches and (**F**) branch length of Iba1^+^ cells in OPL over time 3-14dpl; data show individual average values and mean ± SD, *n* = 5, **p < 0.01 (2-way ANOVA, Tukey’s multiple comparisons test). IPL = inner plexiform layer; OPL = outer plexiform layer

Previous research has shown that SCFA possess anti-inflammatory properties, for example, inhibiting inflammatory cytokine production by microglia and myeloid cells [32–34]. To further investigate the impact of SCFA treatment on retinal microglia, we isolate retinas from control and SCFA treated animals (males and females) after laser-induced CNV, and prepared retinal flat mounts for immunofluorescence, with Iba1 as microglia/myeloid cell marker (Fig. 2A-B). Strong autofluorescence, signal saturation, and frequent tissue disruption at the lesion core made consistent quantification unreliable. To avoid bias from selectively preserved lesions, we chose peri-lesion regions for reproducibility. Isolectin-B4 staining was used to identify blood vessels and discriminate the retinal layers where microglia mainly reside, i.e., inner plexiform layer (IPL, containing large vessels) and outer plexiform layer (OPL, containing small vessels) (Fig. 2B). Overall, the density of Iba1^+^ cells increased from 3 to 7 dpl, and then decreased in both sexes and retinal layers by 14 dpl (Fig. 2C-D). The density of Iba1^+^ cells at 3 and 7 dpl in the OPL in the untreated group was higher in males than in females (Fig. 2D). The SCFA treatment notably prevented the increase of Iba1^+^ cells density at 7dpl in male but not in female retinas in both layers. SCFA treatment in females led to the rise in Iba1^+^ cell density in the OPL at 3 dpl. This coincides with the reduction in the IR, BAF and FA parameters at 3dpl in females by SCFA (**Fig.****S2****A-C**). Since the effect size of microglia density was dominant in the OPL, we further assessed the morphology of Iba1^+^ cells in the OPL as an additional readout for the microglia state (Fig. 2E). At 7 dpl, SCFA treatment increased the number of cellular branches per cell in males (Fig. 2F) and increased the branch length in females (Fig. 2G) indicating a higher ramification index in both sexes.

Altogether, SCFA treatment promoted an increase of Iba1^+^ cells at 3dpl in females, prevented the increase of Iba1^+^ microglia density at 7dpl in male retinal layers, and promoted higher ramification in both sexes. The dynamics in microglia density coincided with a protective effect of SCFA in males and females at different time points after the laser lesion.

### Sex-specific transcriptional differences upon laser-induced CNV

**Fig. 3:**
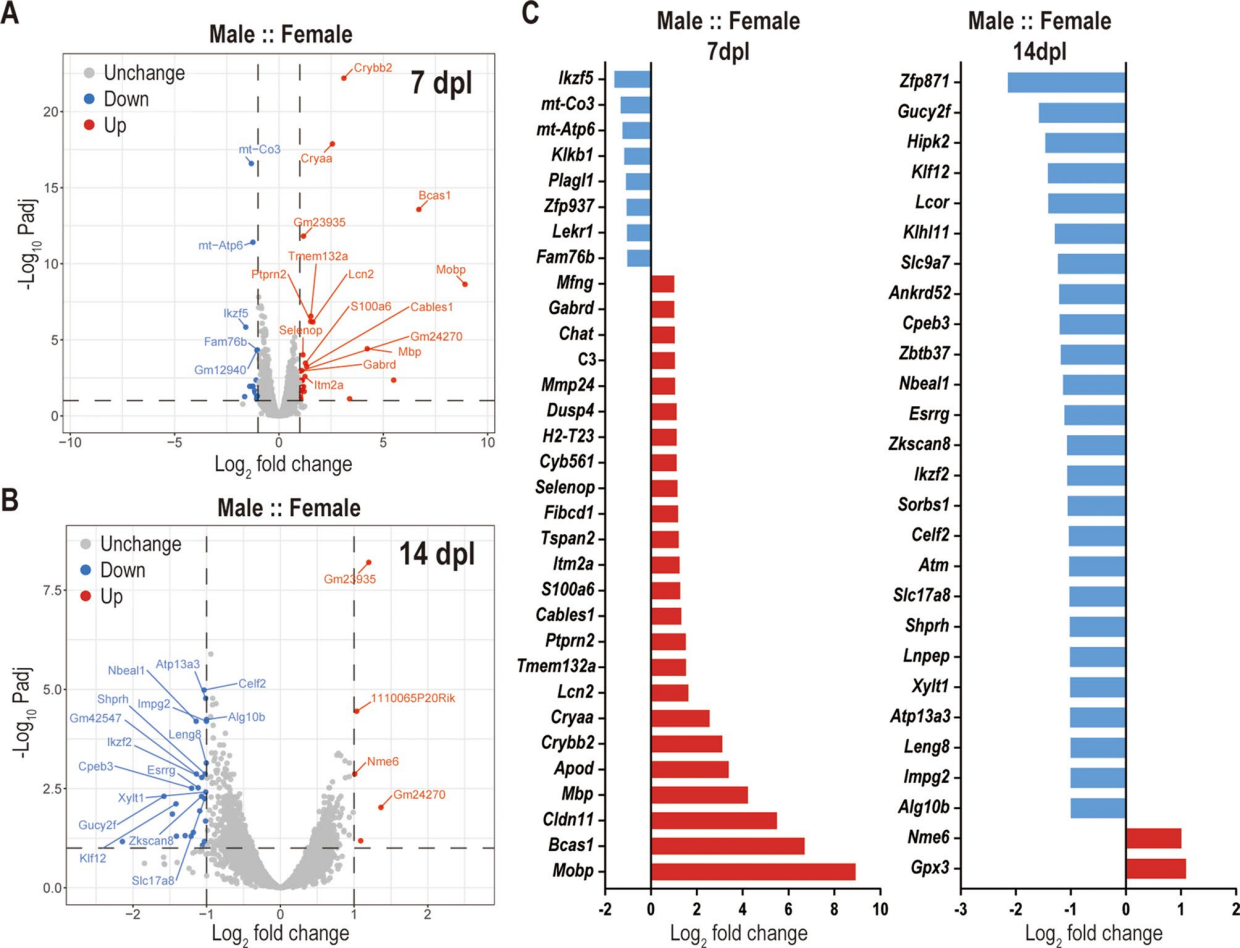
Sex-specific transcriptional differences upon laser-induced CNV. Bulk RNA sequencing analysis from retinas of untreated control groups. (**A**, **B**) Volcano plot of DEGs between male and female control mice, at 7 dpl (**A**) and 14 dpl (**B**) of laser-induced CNV; x-axis shows log2 (|fold change| >1) indicating the expression level differences for each gene between male and female; y-axis shows -Log10 *Padj* (< 0.1) indicating the significance levels for each gene. The top 20 genes based on the ranked padj values were named on the plots; the following X-linked and Y-linked DEGs located on X or Y chromosome are not shown: *Xist*,* Eif2s3y*,* Ddx3y*,* Kdm5d*,* Uty*,* Kdm6a*,* Itm2a and Plp1* at 7 dpl, and *Xist*,* Ddx3y*,* Eif2s3y*,* Kdm5d*,* Uty*,* Kdm6a*,* Xiap*,* Rs1*,* Kdm5c and Eif2s3x* at 14 dpl. (**C**) Bar charts showing DEGs of males over females at 7- and (**D**) 14 dpl; blue bars indicate reduced expression, and red bars increased expression in males compared to females

To further explore sex-specific differences upon laser-induced CNV at the transcriptome level, we performed bulk RNA sequencing of retina samples at 7 and 14 dpl. We would like to emphasize that that all transcriptomic conclusions are hypothesis-generating only. Limitations stem from the restrictions in animal numbers (and therefore n numbers for each group) and the lack of additional PCR verifications. We first evaluated sex differences in the lasered control groups (no treatment) during the development of the CNV model. A total of 53 differentially expressed genes (DEGs) were identified at 7 dpl (Fig. 3A) and 42 DEGs at 14 dpl (Fig. 3B) between sexes, using a fold change > 1 and adjusted p-value (padj) < 0.1. Excluding specific X-linked, Y-linked DEGs, and unidentified sequences, they were reduced to 32 and 27 DEGs, respectively (Fig. 3C). The entire list of DEGs identified in this study is available (Supplementary material 4) as well as the PCA plots (Supplementary material 5 A-D). Males exhibited a significant upregulation of genes related to neuroinflammation, myelination, and synaptic transmission at early time points, whereas females demonstrated greater mitochondrial stability and epigenetic regulation over time. At 7 days, males showed increased expression of C3, Lcn2, and Dusp4, all associated with immune activation and oxidative stress. Additionally, myelin-related genes such as *Mobp, Bcas1, Cldn11,* and *Mbp* were upregulated in males, suggesting a stronger myelination/oligodendrocyte-mediated response. By 14 days, males exhibited decreased expression of *Ikzf2, Esrrg, Sorbs1* and *Nbeal1*, indicating a less effective response to inflammation in males. The reduced expression of *Xylt1 *and *Alg10b*, genes associated with glycosylation processes, could influence cellular interactions and repair mechanisms, affecting the retina’s ability to recover from injury.

Females, on the other hand, exhibited higher expression of mitochondrial function genes such as *mt-Co3* and *mt-Atp6* at 7 days, suggesting a greater reliance on oxidative phosphorylation. By 14 days, genes associated with oxidative stress protection (*Gucy2f and Ankrd52*), as well as genes associated with cell survival and cell-cell signaling (*Atp13a3*,* Klf12*,* Zbtb37* and *Slc17a8)* were upregulated compared to males, highlighting a more sustained metabolic resilience and better response to injury.

Microglia-specific gene expression further demonstrated significant sex differences. Male microglia exhibited enhanced inflammatory activation, considering the comparative higher levels of *C3*, *Lcn2*, and *Dusp4* at 7 days, which are related to a pro-inflammatory microglial phenotype. In addition, *S100a6*, *Selenop*, and *Mmp24* are also genes associated with inflammation, and their expression was higher in males compared to females. Females showed higher expression of *Ikzf5* at 7 days and *Ikzf2*, *Esrrg* at 14 days, suggesting a transition towards reparative roles more efficiently over time.

### SCFA treatment induced sex-specific transcriptional alterations in the retina

Next, we addressed the effects of SCFA treatment on laser-induced CNV on the retinal transcriptome. Analysis of DEGs following SCFA treatment revealed distinct transcriptional responses between males and females at both 7- and 14 dpl, indicating sex-specific regulation of inflammatory pathways, myelination processes, and metabolic function.

#### Males: SCFA effects on gene expression at 7- and 14-days post-lesion

**Fig. 4:**
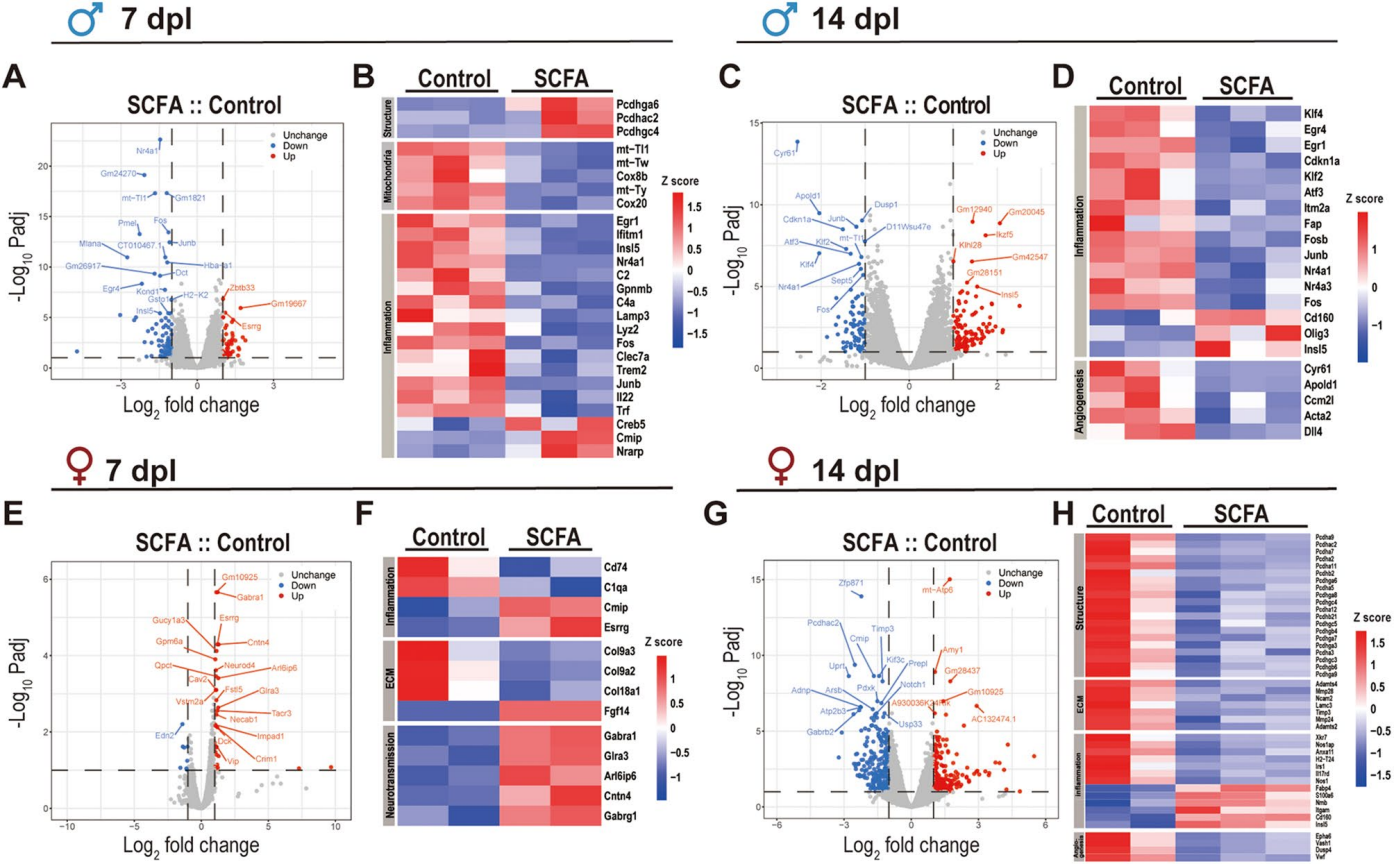
Analysis of genes related to microglial cell activation after SCFA treatment. Bulk RNA sequencing analysis from retinas isolated from animals of the control and SCFA groups at 7 dpl (A, B, E, F) and 14 dpl (C, D, G, H). (A, C, E, G) Volcano plots indicating the distribution of the DEGs and the top 20 genes based on *padj* values were marked. The blue dots indicate downregulated genes by SCFA, the red dots indicate the upregulated genes; the grey spots indicated not differently expressed genes. (B, D, F, H) Heatmaps of selected DEGs visualized according to their expression level (z-score), and clustered according their involvement in the biological process indicated on the left side in grey color

At 7 dpl, SCFA treatment in males led to upregulation of *Pcdhga6*, *Pcdhac2*, and *Pcdhgc4*, which are associated with synaptic organization and neuronal connectivity. Additionally, SCFAs increased expression of *Cmip* and *Nrarp*, suggesting enhanced neuroprotection and axonal remodeling. Conversely, mitochondrial genes such as *mt-Tl1*, *mt-Tw*, and *Cox8b* were downregulated, indicating potential metabolic shifts. Notably, SCFA treatment also affected microglia-specific genes in males, including the downregulation of *Clec7a*, *Trem2*, and *Lyz2*, which are involved in microglial activation and immune signaling. This suggests that SCFAs may suppress excessive microglial activation, thereby modulating neuroinflammatory responses (Fig. 4A-B).

By 14 days, SCFA treatment downregulated key transcription factors including *Klf4*, *Egr4*, and *Egr1*, suggesting suppression of early inflammatory responses. Notably, *Cdkn1a* (a regulator of cell cycle arrest) was also reduced, potentially promoting regenerative capacity. Increased expression of *Olig3* and *Insl5* suggests SCFAs may enhance neurogenesis and metabolic regulation at later time points (Fig. 4C-D).

GO analysis supported these results by revealing an effect of SCFA on neuronal survival and multiple metabolic processes (Supplementary material 5 E, F; Supplementary material 6).

#### Females: SCFA effects on gene expression at 7- and 14-days post-lesion

In females at 7 days, SCFA downregulated *Cd74*, *C1qa*, and *Col9a3*, genes associated with complement system activation and extracellular matrix remodeling. In addition, *Cmip*, *Esrrg*, and *Epha3* were upregulated, indicating enhanced neuroprotective and mitochondrial activity (Fig. 4E-F).

By 14 dpl, SCFA had a striking impact on genes from protocadherin (Pcdh) complexes/clusters, indicating a modulation of chromatin accessibility across an entire genomic region associated with retinal structural development and myelinization (Fig. 4G-H). Most genes linked to ECM remodeling (*Adamts4*, *Mmp28*, *Ncam2* and *Lamc3*), and angiogenesis (*Epha6*, *Vash1*, *Dusp4* and *VwF*) were downregulated in female-SCFA group, while genes related to inflammatory response (*Nos1ap*, *Anxa11*, *H2-T24*, and *CD160*) showed a mix of increased and decreased expression (Fig. 4H).

GO analysis supported these results by revealing an effect of SCFA in extracellular matrix organization, synapse organization, and neurotransmitter secretion and transport (Supplementary material 5 G, H; Supplementary material 6).

These findings highlight that SCFA treatment exerts differential effects on males and females, targeting inflammation resolution, mitochondrial support, and neuronal repair processes in a sex-dependent manner.

### SCFA impact human and murine microglial phagocytosis in vitro

**Fig. 5:**
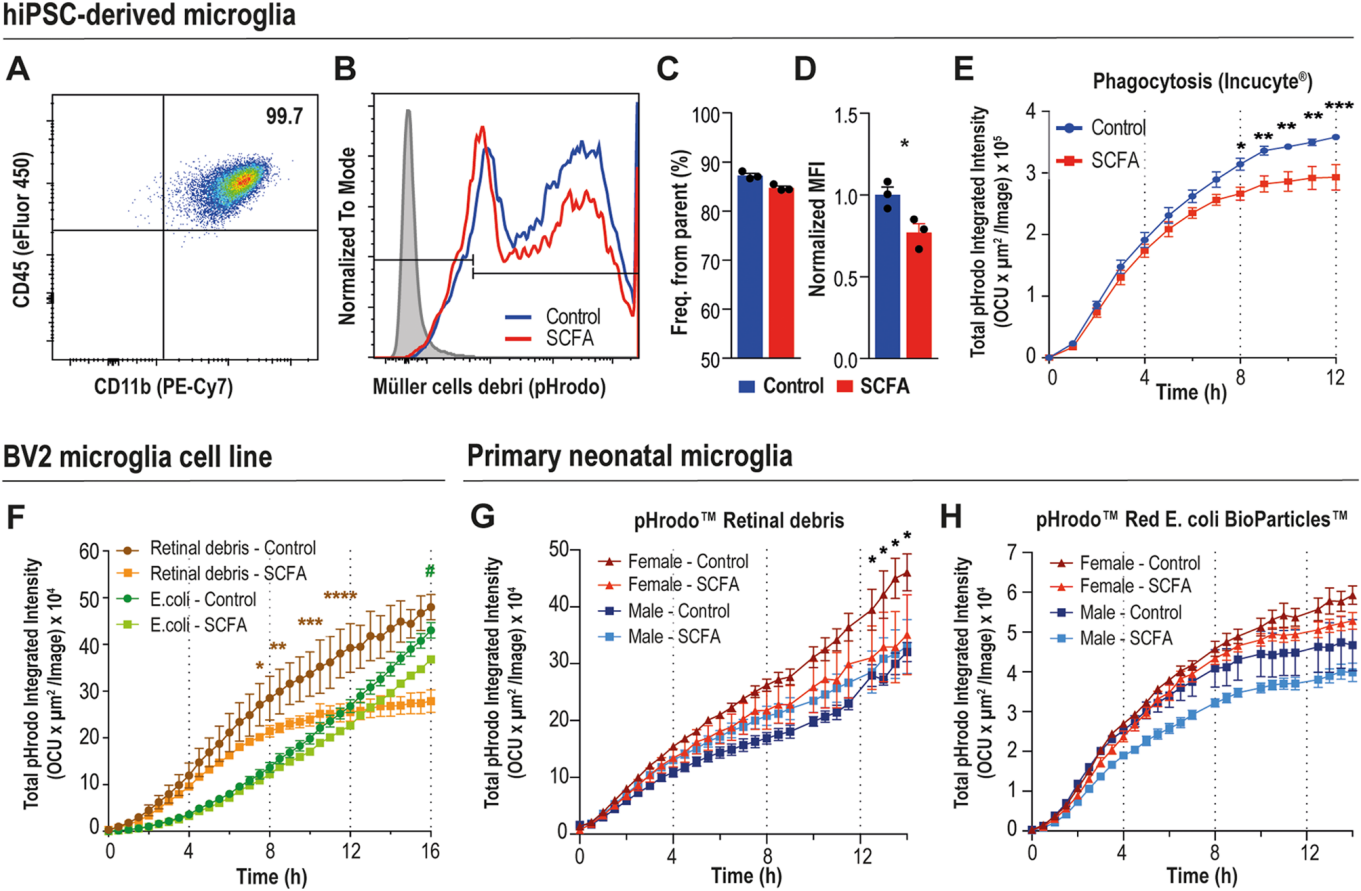
Phagocytic capacity of hiMGLs and BV-2 cells upon SCFA treatment. Microglia phagocytosis was analyzed by flow cytometry (A-D) or monitored over time via Incucyte^®^ (E, F). Cells were preincubated for 4 h in the presence of 300µM acetate, 20µM of propionate, and 10µM of butyrate diluted in the cultivation medium. Then, Müller cell debris, retinal debris or pHrodo™ Red E. coli BioParticles™ were added to the assay. (A) Dot plot shows that hiMGLs express CD45 and CD11b surface markers. (B) Histograms show the engulfment of human Müller cells debris impregnated with pHrodo™ by hiMGLs; grey histogram is a negative control (no baits). (C) Frequency in % of cells derived from parent population (CD45^+^CD11b^+^) that engulfed debris. (D) MFI values of the cells that engulfed debris, normalized to the control group; data are presented as mean + SEM, *n* = 3, *p > 0.05 (unpaired two-tailed t-test). (E) Phagocytosis of pHrodo™-labelled Müller cells debris by hiMGLs; data are presented as mean ± SEM, *n* = 3–6, *p < 0.05, **p < 0.01, ***p < 0.001, (two-way ANOVA with repeated measures, Sídák’s multiple comparisons test). (F) Phagocytosis of pHrodo™-labelled retinal debris or pHrodo™ Red E. coli BioParticles™ by BV-2 microglia cell line; data are presented as mean ± SEM, *n* = 3, *p < 0.05, **p < 0.01, ***p < 0.001, (two-way ANOVA with repeated measures, Tukey’s multiple comparisons test); # *p* < 0.05 represent significance difference for E. coli groups. (G, H) Phagocytosis assay with primary neonatal microglia cells from males and females, performed with (G) labeled retinal debris and (H) E. coli BioParticles™; data are presented as mean ± SEM, *n* = 4–5, *p > 0.05 (two-way ANOVA with repeated measures, Tukey’s multiple comparisons test). OCU = Orange Mean Intensity

Given that SCFA treatment not only modulated microglial density and morphology but also downregulated microglia-expressed genes in the retina, we aimed to assess its direct effects on microglia function. For that, we transitioned from in vivo to in vitro approach. As microglia serve as the primary phagocytes in the central nervous system, we next conducted phagocytosis assays using two distinct in vitro microglial models. First, we employed human induced-microglia-like cells (hiMGLs) derived from a differentiation protocol of human pluripotent stem cells (hiPSCs) [28]. To mimic retinal pathology in the assay, we generated cellular debris from a human Müller cell line and labeled it with pHrodo™. Fully mature hiMGLs were pre-incubated with the SCFA solution and subsequently exposed to the labeled debris. Phagocytic capacity was assessed as an end-point read by flow cytometry (Fig. 5A-D, Supplementary material 7 A). In addition, we monitored real-time phagocytosis using the Incucyte^®^ platform (Fig. 5E). Our data showed that SCFA treatment reduced phagocytic capacity of microglia over time. Furthermore, we used the BV-2 microglial cell line and primary microglia cultures as murine corresponding assays, with two phagocytic baits: retinal debris and pHrodo™ Red E. coli BioParticles™, both labeled with pHrodo™. Cells were pre-incubated with SCFA solution, and real-time phagocytosis was assessed with the IncucyteⓇ platform (Fig. 5F-H, Supplementary material 7B-D). SCFA treatment reduced BV-2 phagocytosis of both baits, with a pronounced effect on retinal debris engulfment (Fig. 5F). Likewise, SCFA reduced retinal debris phagocytosis in primary female microglia, but not in male cells (Fig. 5G). In contrast, SCFA had no significant effect in phagocytosis of E. coli BioParticles™(Fig. 5H). Of note, in both bait settings female microglia showed increased phagocytosis compared to males, a previously described characteristic of female macrophages and neutrophils [35]. Altogether, our results indicate that human and mouse microglia showed reduced phagocytosis upon SCFA treatment, which strengthens the evidence that SCFA influences microglia in the context of retinal pathology.

## Discussion

Sex-specific differences in AMD development and therapeutic response have long been overlooked, as has the role of gut microbiota metabolites in the prevention and treatment of retinal disorders. In this study, we first examined whether male and female mice developed the AMD model similarly. Second, we investigated whether a mixture of SCFAs (acetate, propionate, and butyrate) ameliorate the disease and whether sex-specific differences emerged following SCFA supplementation. Our findings revealed that the CNV model varies between sexes and that SCFA intervention provides protective, yet distinct, effects in males and females, influencing disease progression and modulating retinal cells at both transcriptional and functional levels. However, further studies are necessary, as our current findings—based on bulk RNA sequencing combined with in vivo imaging and cellular analyses—should be considered exploratory.

Without treatment, we observed no significant differences in IR and FA parameters—proxies for CNV size and leakage area—between sexes. We like to emphasize that our ex vivo focus was on the retina – not the choroidea. Thus, we use the in vivo images as a proxy for CNV size and leakage. Note, that other studies use choroidea flat mount to investigate CNV size and leakage ex vivo instead of in vivo imaging. Female mice exhibited less inflammation, as indicated by lower in vivo BAF values compared to males. Differences in the findings between our study and others may arise due to variations in mouse strains, experimental protocols, time points evaluated, housing conditions, and, importantly, the age and sex of the animals used. Previous studies on sex differences in CNV have reported conflicting results, with one study suggesting that female mice develop more severe CNV than males [36], while another study highlighted the role of estrogen and age in these differences [37]. In our dataset, estrus cycle was monitored by vaginal inspection (not shown), and estradiol levels in serum samples that indicated no difference in females from control and treated groups (Supplementary material 8). It is possible that subtle differences may already emerge in younger animals under specific conditions, such as differences in inflammatory responses, hormonal levels, or genetic background. Further studies are warranted to systematically evaluate how age and experimental conditions influence sex-dependent effects in the CNV model. The observed discrepancies underscore the complexity of age- and sex-related differences in CNV studies and highlight the need for standardized protocols to better compare findings across studies.

In females, SCFA reduced lesion hyper-reflectiveness and vascular leakage at 3dpl after the lesion, and significantly lowered inflammatory infiltration at 3 and 14 dpl. In males, SCFA led to a reduction in inflammatory infiltration at 14dpl, but still it had no effect on vascular leakage or lesion hyper-reflectiveness. These findings suggest that SCFA treatment differentially modulate CNV development affecting females predominantly at early time points, and males later after the laser-lesion.

The reduction of inflammation revealed by the BAF parameter at 14dpl in males was accompanied by a reduction of microglia density in the OPL and IPL retinal layers and increased microglia ramification upon SCFA at 7dpl. Thus, the number of retinal microglia in males and females is modulated by SCFA at different time points and directions, coinciding with beneficial effects on CNV parameters. The temporal association between microglial changes and CNV modulation is correlative, not causal. Retinal microglia have been negatively related to AMD pathology, as their accumulation in sites of retinal degradation accelerates drusen body formation, photoreceptor degeneration, and pathological neovascularization [38–40]. Our data imply that SCFA modulate microglia density in vivo “as needed” promoting a slight increase in microglia density in females at 3dpl and preventing a detrimental increase at 7dpl in males. This is consistent with the idea that microglia can be either beneficial or detrimental, depending on the stage of the disease [41]. In our in vitro experiments, SCFA reduced the phagocytic capacity of both human and mouse microglia, revealing a direct effect on these central microglia function. By reducing microglial phagocytosis at the right time, and taking the individual’s sex into account, it may be possible to preserve RPE integrity, limit synaptic loss, and decrease inflammation-driven neovascularization. Baseline sex differences in gene expression revealed that indeed males exhibit dysregulated inflammatory response and a greater reliance on myelin repair, while females maintain better mitochondrial resilience and epigenetic flexibility. These intrinsic differences set the context for how SCFA treatment differentially influences recovery in each sex. The heightened immune activation in males, characterized by increased expression of *C3*, *Lcn2*, and *Dusp4*, suggests that their microglia are in a more reactive state post-lesion, which may contribute to prolonged neuroinflammation. SCFA treatment appeared to counteract it by downregulating microglia-associated genes such as *Clec7a*, *Trem2*, and *Lyz2*, thereby reducing excessive microglial activation and promoting a more controlled immune response. *Clec7a*, *Trem2*, and *Lyz2* are key regulators of microglia phagocytosis [42–44], complementing our in vitro observation of phagocytosis reduction by SCFA. This modulation could be critical for males, as unresolved inflammation is often detrimental to recovery. This also aligns to our findings at the cellular level, which showed that SCFA reduced microglia density at its 7 dpl peak in males only. These findings are in line with research showing that SCFAs regulate neuroinflammation by decreasing microglial activation and inflammatory gene expression, therefore enhancing neuroprotection [34, 45].

In contrast, females demonstrated a more balanced immune response at baseline, along with a stronger mitochondrial advantage, as reflected in the higher expression of *mt-Co3* and *mt-Atp6*. SCFA treatment further enhanced mitochondrial efficiency in females by upregulating *mt-Co2*, *mt-Atp6*, and *Cox8b*, reinforcing their metabolic advantage. This difference in mitochondrial response suggests that while males may require more direct intervention to limit neuroinflammation, females benefit more from metabolic support that sustains neuronal function. Studies have shown that sex differences in mitochondrial function contribute to variations in neurodegenerative disease susceptibility, with females often exhibiting more robust mitochondrial resilience [46], which we also observe here.

Another mechanistical consideration on the SCFA modulation of microglial phagocytosis relies on the differential responses to different phagocyted particles. Here, SCFA inhibited phagocytosis of retinal debris but not *E. coli* particles, likely pointing towards involvement of distinct “sensing” receptors for bacterial particles (e.g. toll-like receptors, TLRs) and cellular debris (e.g. scavenger receptors). In our dataset, expression of several TLRs in males and females were modulated by SCFA treatment, and varied across time points. Of note, in females, TLR2 and TLR13 genes showed altered expression in more than one sample, suggesting SCFA may influence microbial sensing pathways. In contrast, scavenger receptors genes (e.g. *Cd36*,* Mertk*,* Msr1*,* Cd68*,* Scarb1*) showed minimal transcriptional changes in female retinas. In males, only *Scarb1* at 14dpl showed a statistically significant increase (padj = 0.039), while others like *Mertk* and *Msr1* remained unchanged or modestly downregulated without significance. In females, none of these genes reached statistical significance after correction, and there was no consistent downregulation across samples. Overall, although the exploratory character of these data, it might suggest that SCFA modulation of phagocytosis is not mediated by transcriptional alterations of scavenger receptors genes.

Another key sex-specific effect of SCFAs indicates involvement of neuroplasticity and myelination. Males exhibited upregulation of protocadherin-associated genes such as *Pcdhga6*, *Pcdhac2*, and *Pcdhgc4* following SCFA treatment, suggesting increased synaptic remodeling and neuronal plasticity. In contrast, females showed downregulation of *Col9a3* and *Col18a1*, indicating a shift in ECM composition that may optimize neuronal regeneration. Later, 14 DEGs belonging to the Pcdh-α cluster were further reduced in treated female mice, suggesting that SCFA treatment is likely involved in the epigenetic regulation of these gene clusters, which are related to structural changes and myelination [47]. These findings indicate that SCFAs facilitate recovery through different mechanisms: promoting structural support in males, while fine-tuning metabolic pathways and inflammatory control in females.

SCFA treatment also influenced epigenetic and neurogenesis pathways in a sex-dependent manner. In males, upregulation of *Olig3* and *Insl5* at 14 days suggested enhanced neurogenesis and metabolic balance, while in females, early upregulation of *Cmip*, *Esrrg*, and *Epha3* at 7 days implied that SCFAs promote neuronal survival and mitochondrial biogenesis sooner in the recovery process. This timing difference may reflect a fundamental disparity in how males and females adapt to injury, with females potentially activating repair mechanisms earlier than males. These findings are supported by the early effects of SCFA on CNV and leakage size in females.

**Fig. 6:**
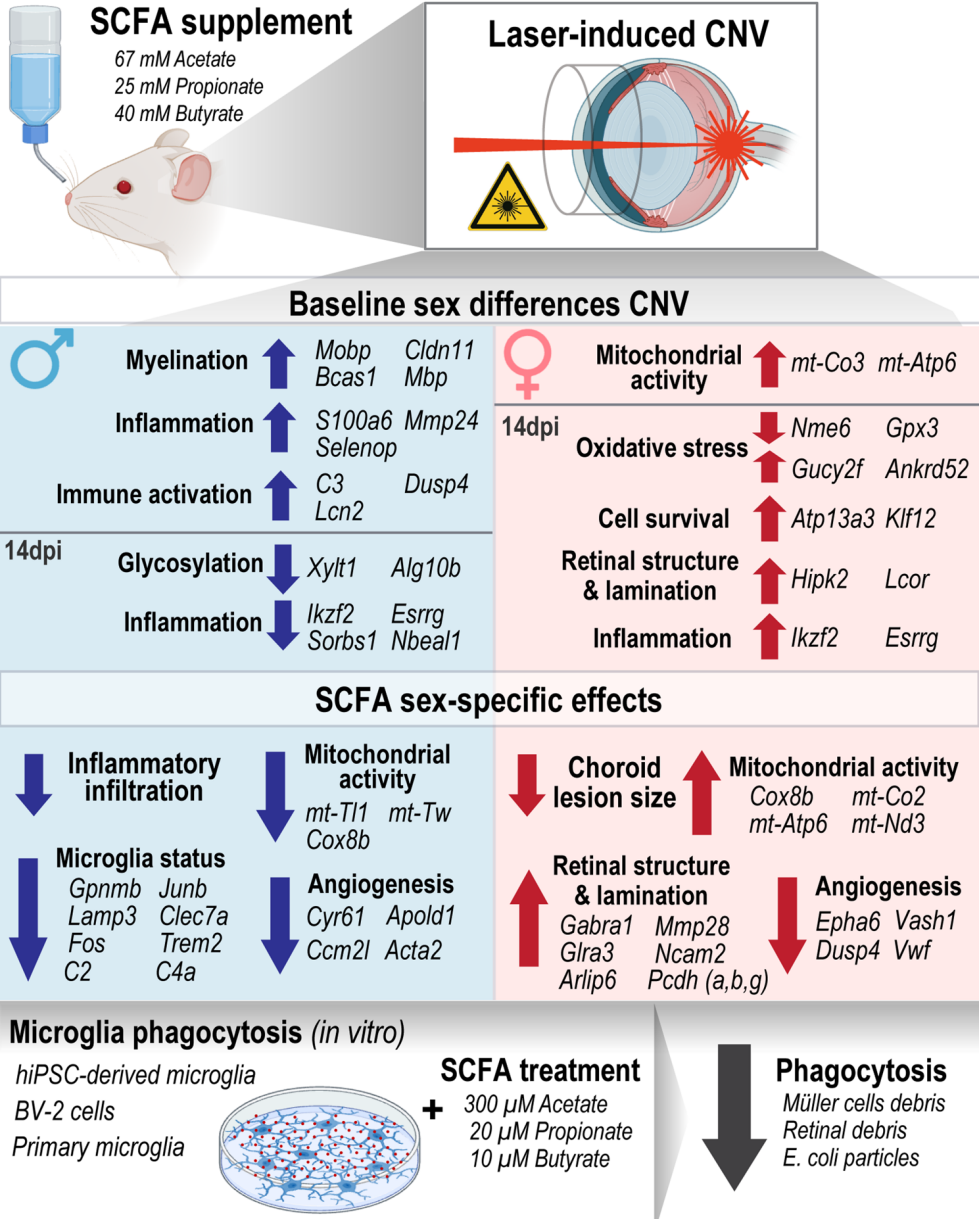
Graphical summary. The figure summarizes the main findings

The overall effects of SCFA treatment underscore the importance of considering biological sex as a key factor in therapeutic strategies for neuroprotection. Males, with their predisposition to heightened inflammation, appear to benefit more from SCFA-driven immune regulation, whereas females, with their stronger baseline mitochondrial function, exhibit enhanced metabolic resilience following SCFA treatment (Fig. 6). These findings suggest that sex-specific therapeutic approaches should be considered when developing interventions targeting neuroinflammation and recovery after retinal injury. Understanding the molecular mechanisms underlying these differences is critical for advancing precision medicine in neuroinflammatory disorders.

## Electronic supplementary material

Below is the link to the electronic supplementary material.

Supplementary Material 1

Supplementary Material 2

Supplementary Material 3

Supplementary Material 4

Supplementary Material 5

Supplementary Material 6

Supplementary Material 7

Supplementary Material 8

## Data Availability

No datasets were generated or analysed during the current study.

## Abbreviations

AMD: Age-related macular degeneration
BSA: Bovine serum albumin
BSA: bovine serum albumin
CD: cluster of differentiation
CNV: Choroidal neovascularization
DEGs: Differentially expressed genes
DMEM: Dulbecco’s Modified Eagle Medium
DPBS: Dulbecco’s Phosphate-buffered saline
E. coli: Escherichia coli
ECM: Extracellular matrix
EDTA: Ethylenediaminetetraacetic acid
FBS: Fetal bovine serum
HEPES: 2-[4-(2-hydroxyethyl)piperazin-1-yl]ethanesulfonic acid
hiMGLs: Human microglia-like cells
hiPSCs: Human induced pluripotent stem cells
HPCs: Hematopoietic progenitor cells
IL-34: Interleukin-34
IPL: Inner plexiform layer
M-CSF: macrophage colony-stimulating factor
MEM: Eagle’s minimal essential medium
nAMD: Neovascular AMD
OCT: Optical coherence tomography
OCU: Orange mean intensity
OPL: Outer plexiform layer
padj: Adjusted p value
Pen/Strep: penicillin /streptomycin
PFA: Paraformaldehyde
ROI: Region of interest
RPE: Retinal pigmented epithelial
RT: Room temperature
SCFA: Short-chain fatty acids
SEM: Standard error of the mean
SD: Standard deviation
TBS: Tris-buffered saline
TGF-β1: Transforming growth factor beta 1
TLR: Toll-like receptor
VEGF: Vascular endothelial growth factor
