# Supplementary figures and images for: Sex differences on laser-induced choroidal neovascularization and short-chain fatty acid treatment in a mouse model
Source: J Neuroinflammation. 2025 Jul 19;22:188. doi: 10.1186/s12974-025-03508-1 (PMC12276663; doi:10.1186/s12974-025-03508-1)

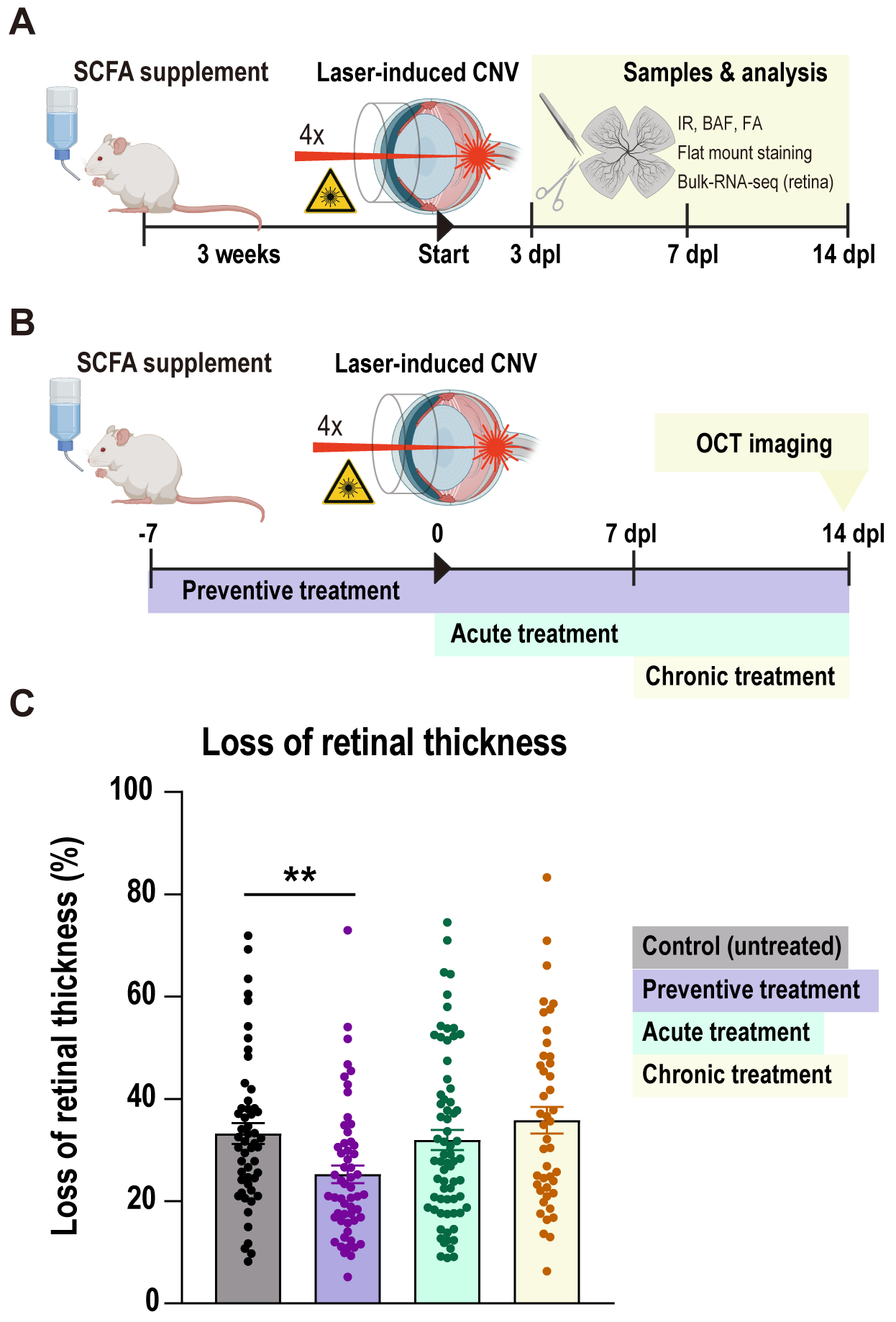

Supplement: Supplementary file 1 — Supplementary Material 1 [file 12974_2025_3508_MOESM1_ESM.tif]

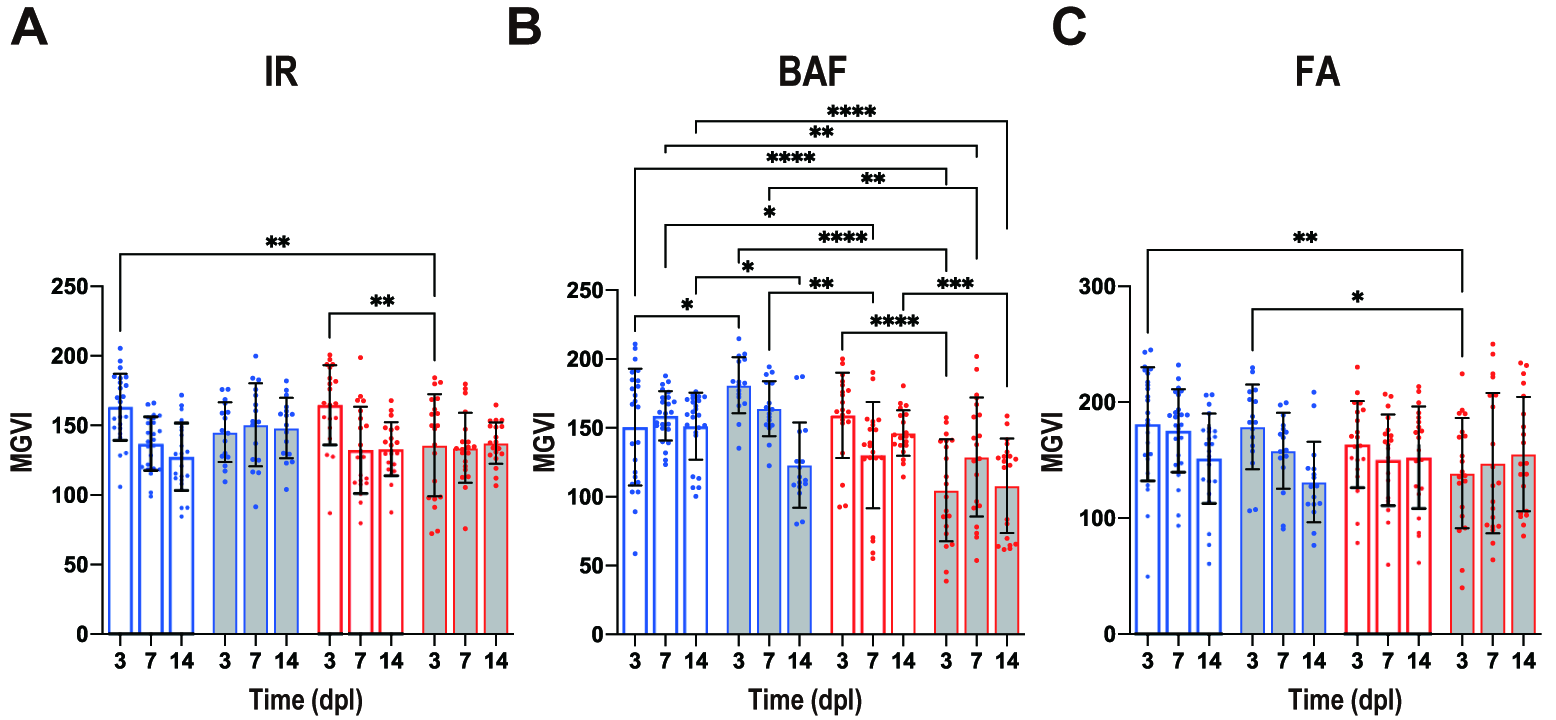

Supplement: Supplementary file 2 — Supplementary Material 2 [file 12974_2025_3508_MOESM2_ESM.tif]

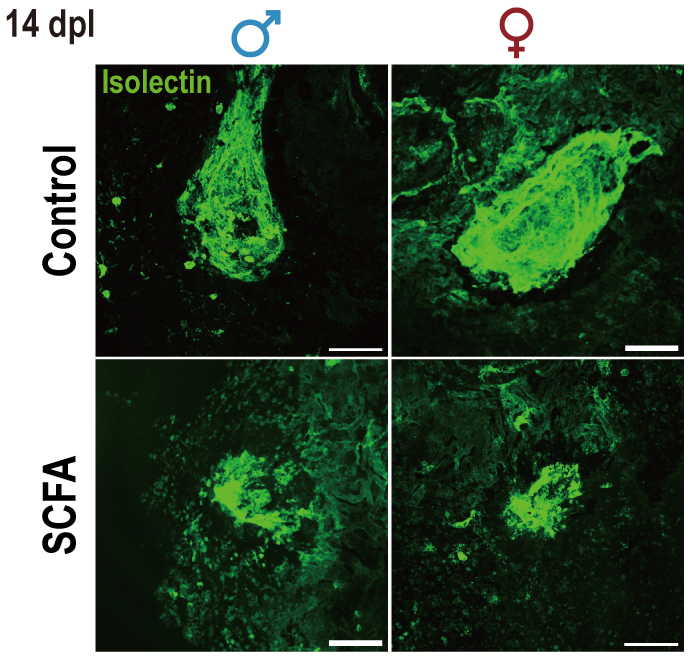

Supplement: Supplementary file 3 — Supplementary Material 3 [file 12974_2025_3508_MOESM3_ESM.tif]

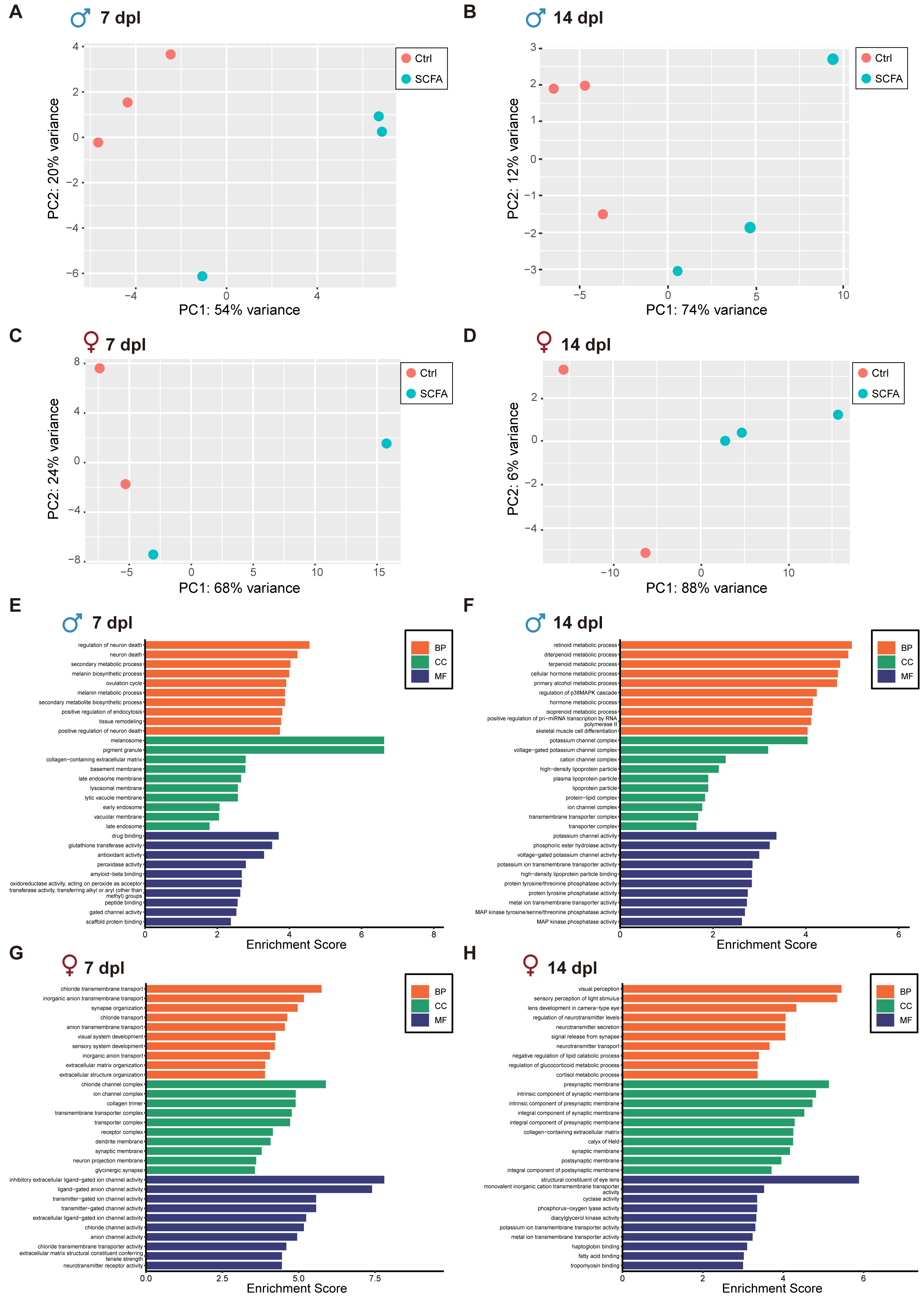

Supplement: Supplementary file 5 — Supplementary Material 5 [file 12974_2025_3508_MOESM5_ESM.tif]

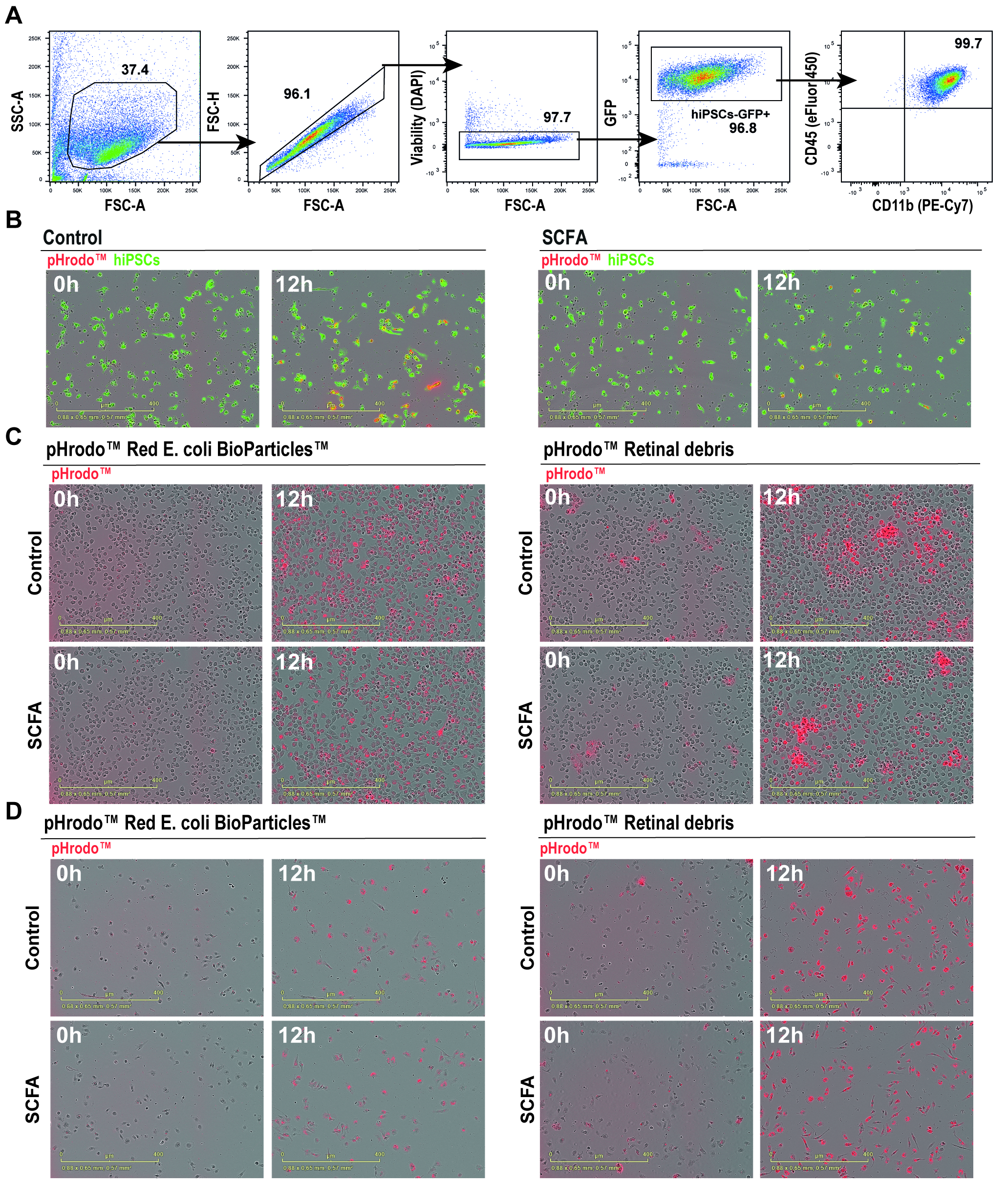

Supplement: Supplementary file 7 — Supplementary Material 7 [file 12974_2025_3508_MOESM7_ESM.tif]

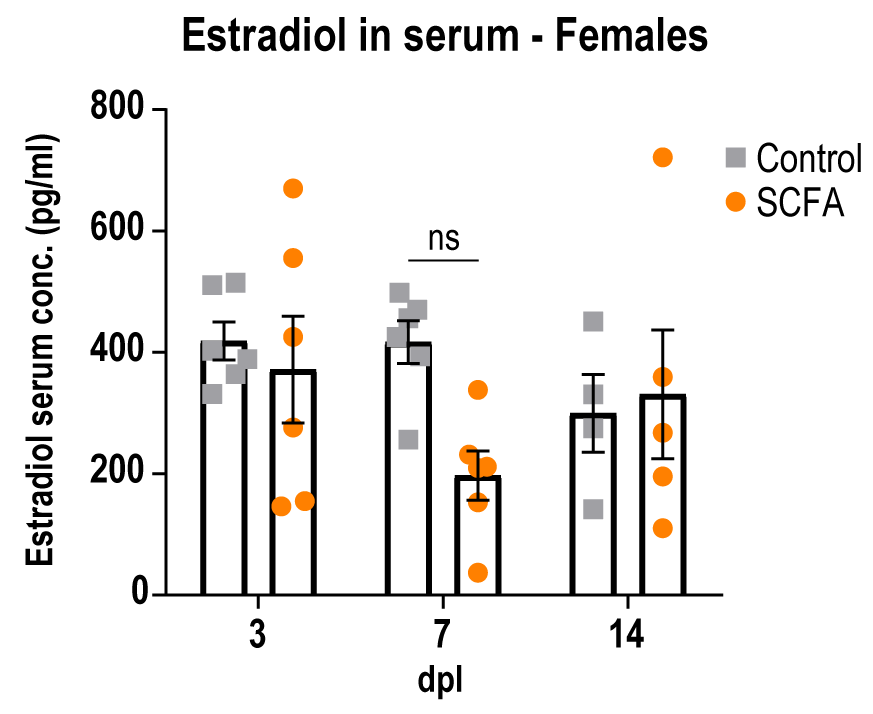

Supplement: Supplementary file 8 — Supplementary Material 8 [file 12974_2025_3508_MOESM8_ESM.tif]
